# Vortex flow in the left atrium in healthy controls and patients with mitral valve regurgitation after atrioventricular septal defect correction: evaluation with 4D Flow MRI and particle tracing

**DOI:** 10.1186/1532-429X-17-S1-Q123

**Published:** 2015-02-03

**Authors:** Emmeline Calkoen, Patrick J de Koning, Rob J van der Geest, Albert de Roos, Jos J Westenberg, Arno Roest

**Affiliations:** 1 Pediatric Cardiology, Leiden University Medical Center, Leiden, Netherlands; 2 Radiology, Leiden University Medical Center, Leiden, Netherlands

## Background

During systole the left atrium serves as a reservoir, in which the inflow from the pulmonary veins is collected and partially organized by vortical flow [Fyrenius et al. Heart, 2001]. Aim of current study was to depict the origin of this vortical flow and evaluate the impact of mitral valve regurgitation on this flow structure.

## Methods

12 healthy controls (age 9-53 years) and 8 patients (age 8-37 years) with a corrected atrioventricular septal defect and a mean mitral valve regurgitation of 25% (range 19-37%) were included. The maximal left atrial volume (LAV) was calculated based on the biplane area-length method from a standardized 2- and 4-chamber view. Whole-heart 4D Flow MRI was performed on a 3T MR scanner with free breathing, three-directional velocity encoding of 150cm/s in all directions, spatial resolution 2.3×2.3×3.0-4.2mm^3^ and 30 phases reconstructed over one cardiac cycle. At end-systole the vortical flow was assessed by manually segmenting the volume of circular flow in the left atrium based on streamline visualization in a stack of slices parallel to the 4-chamber view. 3D-particle tracing was applied in reverse time order, using the defined volume as seeding, to trace back the seed points and quantify the number of particles originating from the left and right pulmonary veins (LPV and RPV).

## Results

In controls mean LAV was 60±35mL and the vortex flow volume at end-systole 8±5mL. Tracing revealed a dominating contribution to the vortical volume originating from the LPV (41±14%), a smaller part from the RPV (17±12%) and a residual part of particles present inside the atrium at the start of systole (42±15%).

**Figure 1:**
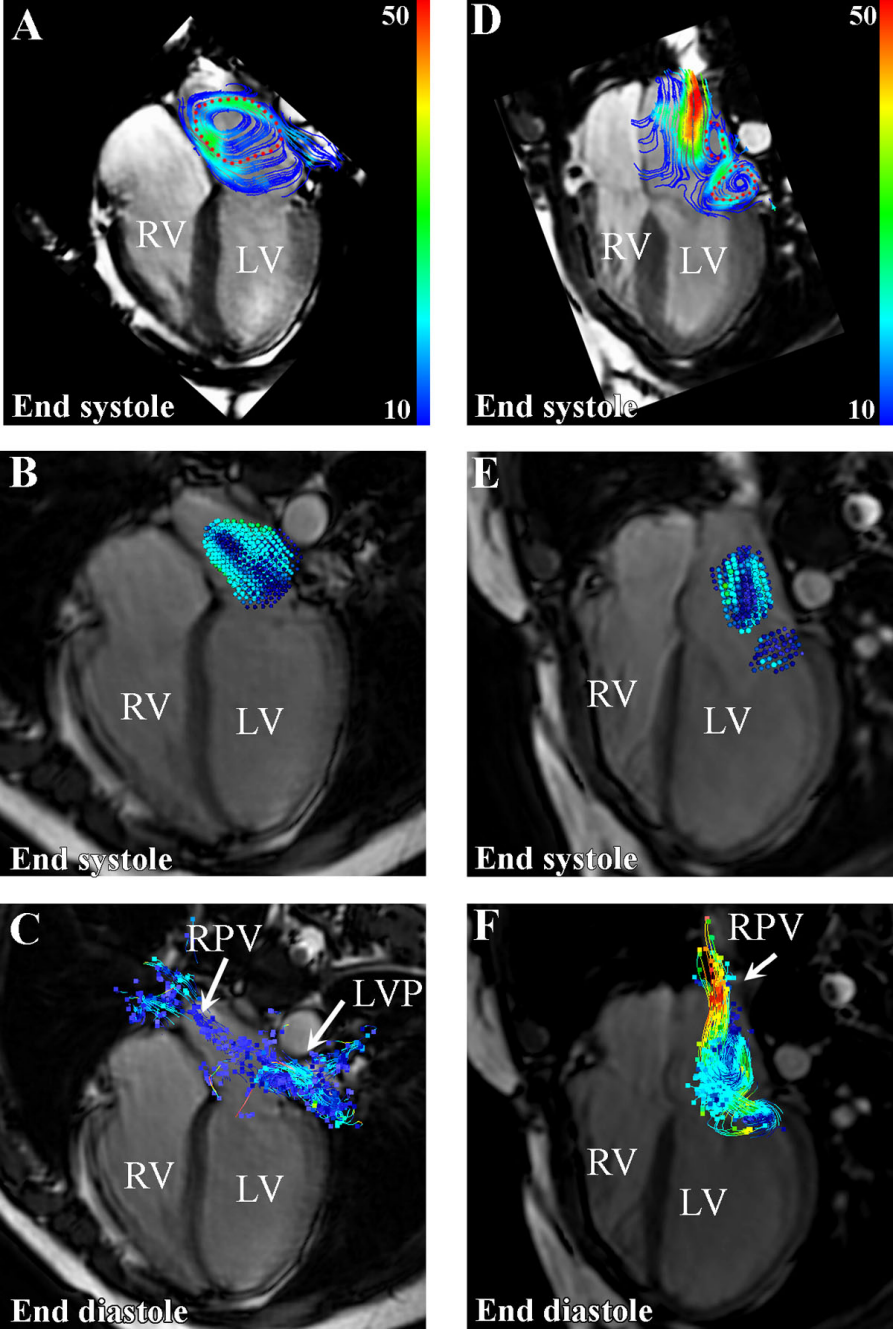
Vortical flow in the left atrium in a control subject (A-C) and patient with laterally directed regurgitation (D-F). In A and D the red dotted contour indicate the segmented region of vortical flow. B and E shows the particle distribution at end-systole. C and F depicts the origin of the segmented vortical flow region as assessed by backward particle tracing.

In patients with mitral regurgitation the LAV was 76±21mL and a complex shape of the vortical flow with variation between subjects was observed (Figure 1). Mean vortical volume at end-systole was 5±4mL, with 13±14% contribution from LPV (difference with controls, p=0.001), 6±6% from the RPV (p=0.049) and a residual part of 81±14% (particles already inside the atrium at start of systole or coming from the ventricle as regurgitation) (p<0.001). Mean contribution ratio between LPV versus RPV was variable including patients with solely contribution from RPV or LPV.

## Conclusions

In normal subjects, the vortical flow inside the left atrium during systole originates from both LPV and RPV, with a higher contribution from the left side. Mitral valve regurgitation disturbs this organized flow, resulting in a reduced contribution of LPV to the vortical flow, potentially leading to less efficient ventricular filling and stasis.

## Funding

E.E. Calkoen is financially supported by a grant from the Willem-Alexander Kinder- en Jeugdfonds, J.J.M. Westenberg is financially supported by a grant from the Dutch Technology Foundation (STW), project number 11626.

